# Fabrication of High-Quality and Strain-Relaxed GeSn Microdisks by Integrating Selective Epitaxial Growth and Selective Wet Etching Methods

**DOI:** 10.1186/s11671-020-3251-0

**Published:** 2020-01-21

**Authors:** Guangjian Zhu, Tao Liu, Zhenyang Zhong, Xinju Yang, Liming Wang, Zuimin Jiang

**Affiliations:** 1 State Key Laboratory of Surface Physics, Department of Physics, Fudan University, Shanghai, 200433 China; 2 Wide Bandgap Semiconductor Technology Disciplines State Key Laboratory, School of Microelectronics, Xidian University, Xi’an, 710071 China

**Keywords:** GeSn microdisk, Molecular beam epitaxy, Selective wet etching, Micro-Raman spectroscopy, Strain relaxation

## Abstract

GeSn is a promising material for the fabrication of on-chip photonic and nanoelectronic devices. Processing techniques dedicated to GeSn have thus been developed, including epitaxy, annealing, ion implantation, and etching. In this work, suspended, strain-relaxed, and high-quality GeSn microdisks are realized by a new approach without any etching to GeSn alloy. The GeSn alloy was grown on pre-patterned Ge (001) substrate by molecular beam epitaxy at low temperatures. The transmission electron microscopy and scanning electron microscopy were carried out to determine the microstructures of the GeSn samples. The microdisks with different diameters of Ge pedestals were fabricated by controlling the selective wet etching time, and micro-Raman results show that the microdisks with different dimensions of the remaining Ge pedestals have different extents of strain relaxation. The compressive strain of microdisks is almost completely relaxed under suitable conditions. The semiconductor processing technology presented in this work can be an alternative method to fabricate innovative GeSn and other materials based micro/nano-structures for a range of Si-compatible photonics, 3D-MOSFETs, and microelectromechanical device applications.

## Introduction

Germanium-tin (GeSn), a complementary metal-oxide-semiconductor (CMOS) compatible group IV material, has drawn significant attention in recent years for its applications in electronics and optoelectronics. Alloying more Sn into Ge can improve the carrier mobility as well as change the bandgap from indirect to direct transition [1, 2]. Theoretical work [3–5] and photoluminescence-based experiment studies [6–8] show that the indirect-to-direct transition for relaxed GeSn alloy occurs at Sn content no less than 6.5%. However, the low (1%) equilibrium solubility of Sn in Ge [9, 10] and the large lattice mismatch (~ 15%) between Ge and α-Sn introduce enormous challenges for the realization of defect-free GeSn alloy with even a few atomic % of substitutional Sn. The use of non-equilibrium growth techniques such as low-temperature molecular beam epitaxy (MBE) [11–15], chemical vapor deposition (CVD) [16–20], and solid-phase epitaxy [21, 22] is in great need.

In the case of GeSn alloy grown on strain-relaxed Ge virtual substrate or Ge substrate, the highest quality of GeSn is expected to be achieved in the pseudomorphic, or fully-strained condition which can avoid the formation of misfits and threading dislocations. Nevertheless, such GeSn alloy is compressively strained (~ 0.15% per 1% Sn) and this epitaxy-induced strain negates the effect of alloying Sn with Ge for bandgap conversion. As a result, a much higher Sn content of 17% for pseudomorphic GeSn epi-layer on Ge (001) [23] is required to achieve the direct bandgap, leading to extremely high challenges for epitaxy and low material quality. Therefore, manipulation of the strain without sacrificing the quality of crystal of the GeSn epi-layers becomes a very important issue. Selective removal of the stress-inducing Ge virtual substrate or Ge substrate under the GeSn epi-layers to form partially suspended microstructures is a promising technique to overcome the compressive strain in the GeSn films. For example, suspended GeSn microdisks with a supporting pillar in the center were fabricated [24–28]. The structure can not only relax the compressive strain in GeSn layer through elastic deformation at free surfaces but also confine optical models near the edge of the microdisk due to the strong refractive index contrast between the GeSn and the surrounding medium (air), such as whispering-gallery-mode [16, 25]. Up to now, only one method to prepare GeSn microdisk through the post-growth optical lithography and top-down etching of the two-dimensional GeSn film is reported [16, 24, 29, 30]. However, the process may suffer thermal mismatch effects during the post-growth etching process, which will lead to a degraded material quality of GeSn microdisks. Recently, P.Ponath et al. reported the selective area growth of highly crystalline *c*-axis oriented BTO [31], which inspired us for the fabrication of GeSn microstructures. By depositing directly GeSn microdisks at last step on a pre-patterned SiO_2_/Ge substrate and then selectively etching away the Ge substrate following the sacrificial SiO_2_ layer removal, suspended GeSn microdisks can be fabricated without the conventional and complex post-growth etching process. Such a method, if feasible, can avoid the aforementioned problems and thus obtain a higher-quality and strain-relaxed GeSn microstructure. Moreover, it is also a very promising method which can achieve arbitrary patterns with high accuracy and a good aspect ratio, especially for three-dimensional integration of complex device structures which need an exact thickness control of layers.

In this paper, GeSn microdisk structures were fabricated successfully by a new method. It is the first time to prepare GeSn microdisks by combining selective epitaxial growth with a simple step of selective wet etching. The thickness, the Sn concentration, and the crystal quality of the GeSn epi-layer were characterized by high-resolution transmission electron microscopy (HRTEM) and secondary ion mass spectrometry (SIMS). Scanning electron microscopy (SEM) and micro-Raman spectroscopy (μ-Raman) were used to gain insights into the microstructures of the fabricated GeSn microdisks. Room temperature (RT) μ-Raman results show that the microdisks with different dimensions of the remaining Ge pedestals have different extents of strain relaxation. The compressive strain of microdisks is almost completely relaxed under suitable conditions. This method to fabricate microdisks without the need to etch the GeSn itself is beneficial to obtain relaxed and high-quality GeSn and other materials nanostructures.

## Methods

### Materials

The Ge wafers were purchased from AXT company. Acetone, isopropyl alcohol, hydrofluoric acid, ethyl alcohol, hydrogen peroxide, ammonia aqueous and potassium hydroxide were supplied by Sinopharm Chemical Reagent (China). Deionized H_2_O (18.2 MΩ·cm) was obtained from an ultrafiltration system (Milli-Q, Millipore, Marlborough, MA).

### Preparation of the Patterned Ge Substrate

**Fig. 1:**
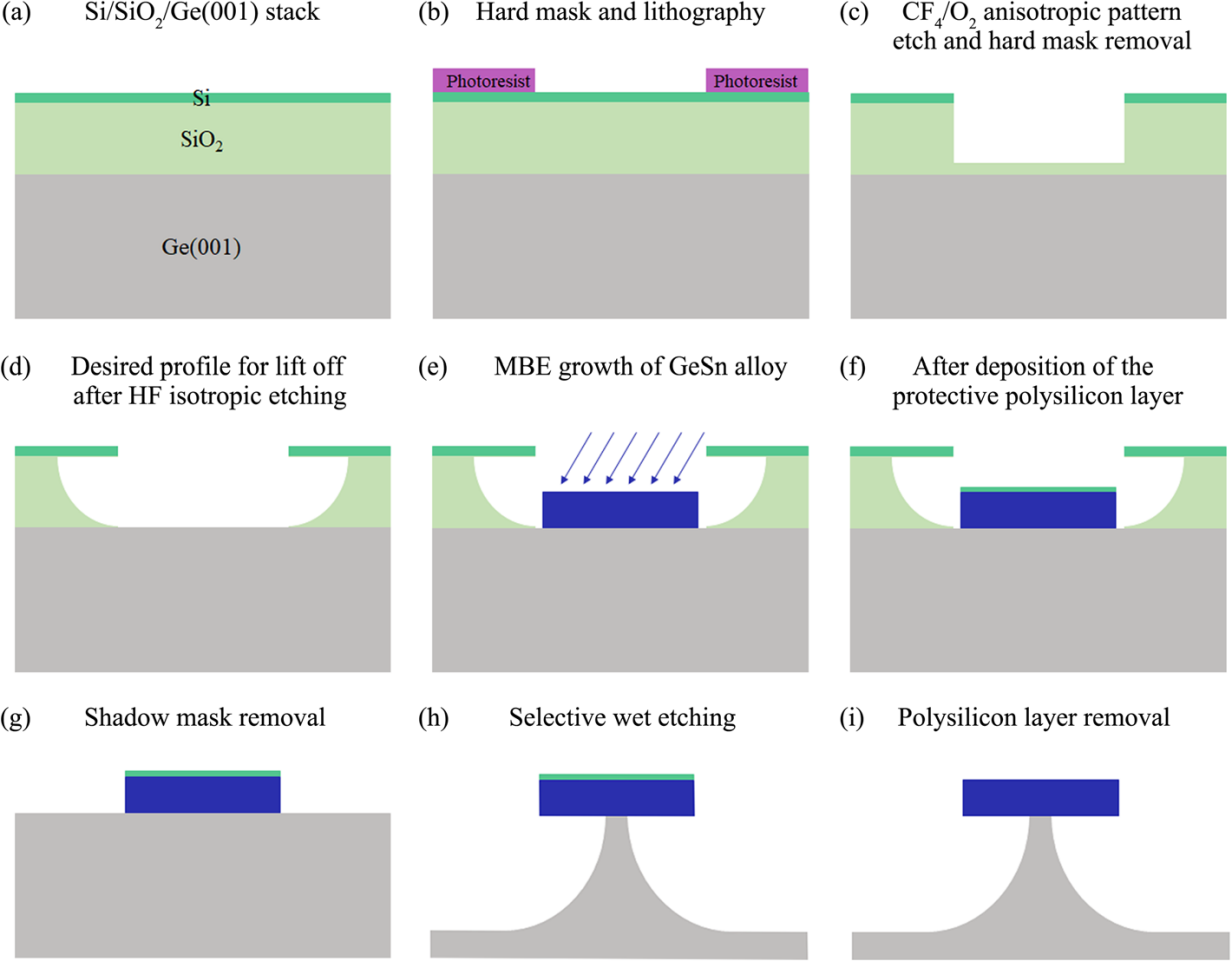
The cross-sectional schematics illustration for the fabrication of the suspended GeSn microdisks

Ge (001) wafers (n-type, 0.025–0.031 Ωcm) were first immersed in acetone and isopropyl alcohol for 3 min and then chemically cleaned using a diluted hydrofluoric acid solution (HF:H_2_O = 1:20) for 20 s at RT. This was followed by a rinse in running deionized H_2_O (DI–H_2_O). The cleaning procedure is of importance, especially the HF treatment to peel-off the native oxide layer and to ensure that the Ge surface is clean and close contact with the next SiO_2_ layer. In this case, the lift off profile is achieved by the deposition of a Si/SiO_2_ composite layer. Then the wafers were dried by blowing dry N_2_ and quickly loaded into the ultrahigh vacuum (UHV) chamber of plasma-enhanced chemical vapor deposition (PECVD) and annealed at 300 °C for 20 min to completely outgas. Then, a 300 nm SiO_2_ layer was deposited at the same temperature by PECVD followed by a deposition of 50 nm undoped polycrystalline Si by magnetron sputtering at RT, as shown in Fig. 1a. Circular openings in the Si/SiO_2_ composite layer are patterned by using standard photolithography technique (Fig. 1b) and two-step etching processes (Fig. 1c, d).

In detail, prior to pattern the Si/SiO_2_/Ge wafer, the wafer was cleaned by acetone, ethyl alcohol, and DI-H_2_O, then blow-dried with N_2_ and pre-baked in an oven at 90 °C. Thereafter, it was spin-coated by AZ5214 photoresist using a spin-coater at a speed of 4000 r/min for 30 s and then placed on a hotplate for soft baking at 90 °C for 2 min. The plate-making machine (Heidelberg, uPG501) was then utilized to create circle-shape pattern arrays on the Si/SiO_2_/Ge stacks. The circular patterns are fixed at 5 μm apart from each other, while the diameter ranges from 3 to 5 μm. The patterns were then transferred to the bottom SiO_2_ layer by two-step etching. The anisotropic dry etching by reactive ion etching with a mixed gas of CF_4_ and O_2_ was firstly employed to etch Si/SiO_2_ layers and stopped with about 20 nm SiO_2_ left. Then dissolving the photoresist and wet etching using diluted HF at RT was hired to remove the left SiO_2_ layer to both expose the germanium in the circular openings and also to etch laterally under the defined polysilicon edge to create an overhang which is the desired profile for lift off.

### GeSn Growth and Microdisk Fabrication

For the GeSn thin film deposition, a solid source MBE system (Riber SSC) with a base pressure of 2 × 10^−10^ Torr was used [32]. An electron beam evaporator and a pyrolytic BN effusion cell using high-purity solid sources are equipped for Ge and Sn evaporation in the MBE system. A quartz crystal monitor was used to calibrate the deposition rate. Before loading the patterned substrates into the UHV system, the substrates were cleaned by O_2_ plasma to remove any organic residue induced by dry etching. Followed by rinsing in acetone, isopropyl alcohol, and diluted HF, the substrates were transferred into the MBE chamber for the GeSn growth. After thermal desorption at 450 °C for 15 min, the substrates were in situ cooled down to 150 °C for the GeSn alloy layer growth with a Ge growth rate of 0.5 Å s^−1^ and a controlled Sn flux as the dopant source. This growth temperature is low enough to efficiently restrain the Sn surface segregation. In order to prevent oxidation, 10 nm Ge cap layer was finally deposited at the same temperature.

**Fig. 2:**
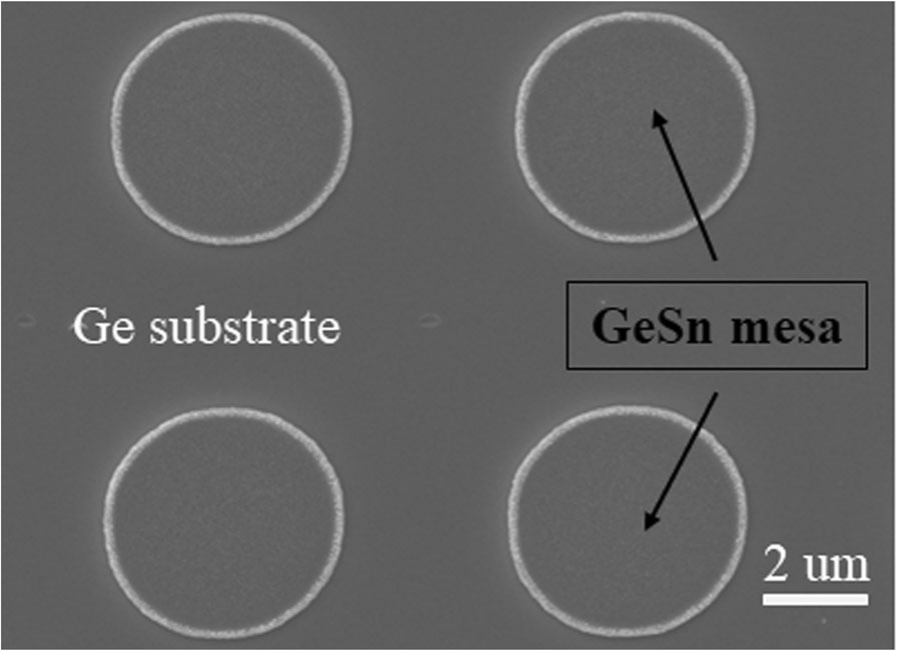
Top-view SEM image of isolated 5 μm diameter GeSn circular mesas after removal of the shadow mask

After growth, GeSn alloys with a nominal Sn concentration of 4% and 10% will be used for microdisk fabrication. An additional 30-nm-thick polysilicon layer was then deposited on the top of the samples at RT using magnetron sputtering to protect the GeSn layer from the following etching. Then, the sacrificial SiO_2_ layer was removed using diluted HF (1:1), which also leads to the removal of the overlaying polysilicon layer and the GeSn film deposited on it. As can be seen in Fig. 2, isolated circular GeSn mesas were revealed after the SiO_2_ layer was removed. However, the periphery of the circular mesa is not so smooth which is bad for optical confine effects. The unexpected situation is caused by the GeSn deposition on the edge of the bottom SiO_2_ layer which arises from the insufficient lateral etching of the bottom SiO_2_ and the off-normal angle (30°) deposition. Subsequently, a simple step of selective wet etching undercuts the circular mesas at RT, resulting in suspended GeSn microdisk structures. The selective etchant (APM) comprises of H_2_O_2_ (31 wt%), NH_4_OH (28 wt%), and DI-H_2_O with a volume ratio of 2:0.5:80 [30]. Finally, the top protective polysilicon layer was removed by KOH. A schematic of the process steps is shown in Fig. 1e–i.

In our method to fabricate GeSn microdisks, the final selective wet etching is important due to the different etch rates for the GeSn materials with different Sn concentrations. The etch selectivity of Ge over Ge_1−x_Sn_x_ will also vary with the Sn concentration. The higher the concentration of Sn in GeSn, the higher the surface coverage of SnO_y_ will be developed [33]. It will reduce the wet etching rate of GeSn and lead to a higher etch selectivity of Ge over GeSn. The previous study has reported that the H_2_O_2_ based wet etch (H_2_O_2_:NH_4_OH:H_2_O = 2:0.5:80), the same with this work, achieves an etch selectivity of Ge over Ge_0.928_Sn_0.072_ of 9:1 [30].

## Results and Discussion

**Fig. 3:**
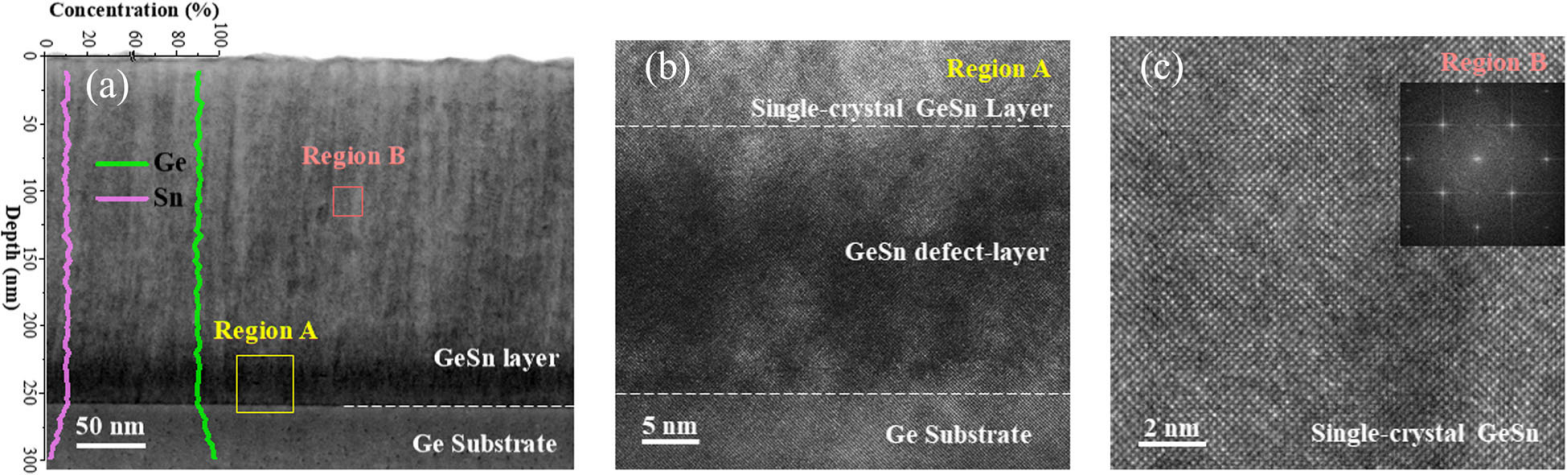
**a** Cross-sectional TEM image of the 5 μm diameter GeSn circular mesa without top polysilicon layer. Inset: SIMS depth profile of the GeSn layer. **b** High-resolution TEM image of the GeSn/Ge interface (region A). **c** High-resolution TEM image for the middle region of the GeSn layer (region B). Inset: FFT pattern of region B

Figure 3a shows the cross-sectional TEM micrograph of the 5 μm diameter GeSn circular mesa without the top polysilicon layer. Figure 3b, c are the high-resolution TEM (HRTEM) micrographs for the regions A and B, corresponding to the GeSn/Ge interface and the middle region of the GeSn layer, respectively. The film thickness of the GeSn layer is about 250 nm (Fig. 3a) below the thickness of the shadow mask. As can be seen in Fig. 3b, defects are mainly localized at the GeSn/Ge interface of 30 nm thickness, resulting in the following single-crystal GeSn growth. The HRTEM image of region B shows clear lattice fringes of the GeSn layer, indicating the alloy is highly crystalline and the fast Fourier transformation (FFT) pattern of region B well matches the diffraction pattern of GeSn as shown in Fig. 3c. In addition, to confirm the Sn concentration of GeSn alloy, SIMS measurement was completed as shown in the inset of Fig.3a. The Sn concentration is 9.8 ± 0.5%, in consistent with the nominal value of about 10%. Apart from that, the distribution of Sn atoms is very uniform in the depth profile of the as-grown GeSn film.

**Fig. 4:**
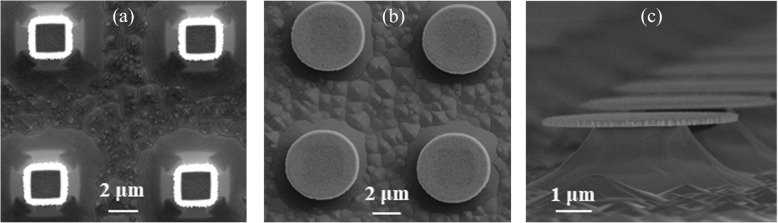
SEM images after selective wet etching. **a** 5 μm diameter Ge_0.96_Sn_0.04_ circular mesas after 510 s etching. **b**, **c** 5 μm diameter Ge_0.902_Sn_0.098_ circular mesas after 510 s etching

The surface structure of the samples was studied by SEM. Figure 4a shows the top view SEM image of the 5 μm diameter Ge_0.96_Sn_0.04_ circular mesas after 510 s selective wet etching by APM solution at RT. It has to be mentioned that the etchants are prepared right before the experiments to avoid the effects of the chemicals aging. The SEM image indicates that not only the Ge substrate but also a part of the GeSn mesa are etched away. The failure to form a disk structure for Ge_0.96_Sn_0.04_ alloy is due to the low concentration of Sn which results in the low etch selectivity of Ge over GeSn. In contrast to the Ge_0.96_Sn_0.04_ samples, the 5 μm diameter GeSn circular mesa sample with higher Sn concentration of 9.8% was also etched by APM at RT. As shown in Fig. 4b, c. c, microdisk structures were formed successfully for Ge_0.902_Sn_0.098_ alloy after 510 s wet etching. Previously, Han et al. [30] reported that they fabricated partly suspended Ge_0.928_Sn_0.072_ microdisks (5 μm in dimension) with about 1.2 μm undercut using the H_2_O_2_ based wet etching with a selectivity of 7:1. However, after the selective wet etching, the edges of their disk become bent and fractured because of strong attractive capillary forces developed between the suspended structure and the substrate as the etchant liquid is removed, which pulls the released microdisk structure into contact with the substrate [24]. But this phenomenon does not occur in our experiments even prolonging the selective etching to nearly completely remove the underneath Ge pedestal. It could be explained by the higher etch selectivity and the thicker GeSn layer of about 250 nm. Interestingly, the pedestals show unexpected facets with different orientations which may be caused by orientation-dependent lateral etching rate during the selective wet etching process.

**Fig. 5:**
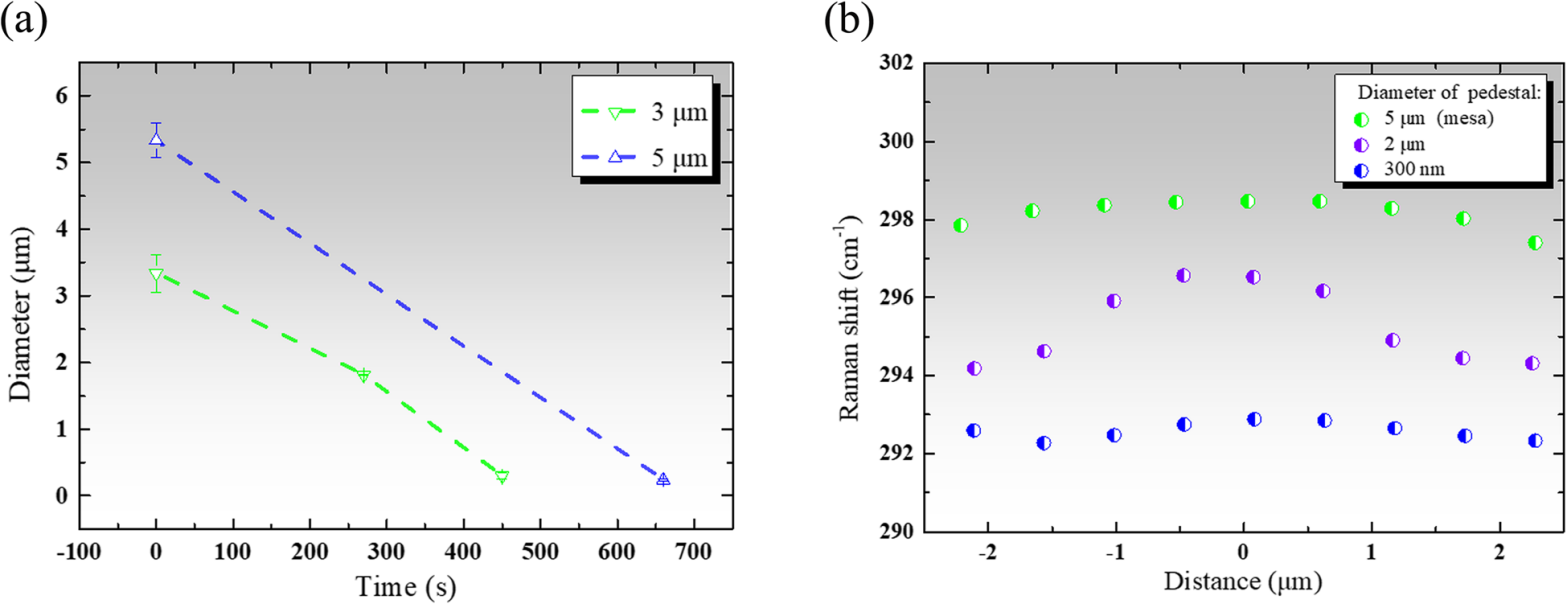
**a** The diameter of the Ge pedestal as a function of selective wet etching time for 3 μm (green dashed line) and 5 μm (blue dashed line) diameters Ge_0.902_Sn_0.098_ disks. **b** Raman shift line scan measurements performed along the diameter of 5 μm Ge_0.902_Sn_0.098_ mesa (green dot) and microdisks with 2 μm diameter Ge pedestal (purple dot) and 300 nm diameter Ge pedestal (blue dot)

In addition, the microdisks with Ge pedestals of different diameters were fabricated by controlling the selective etching time. Figure 5a shows the diameter of the remaining Ge pedestal as a function of etching time in APM. The error bars represent the standard deviation from the data of five different microdisks in the same sample. The similar line tendency indicates that the etch rate of Ge is roughly equal for Ge_0.902_Sn_0.098_ disks with different diameters of 3 μm and 5 μm, and is in agreement with the value of the etch rate from Ref. [30]. Moreover, the minimum diameter of Ge pedestal for standing GeSn microdisk is about 300 nm. The suspended GeSn microdisks will be pulled down by the strong attractive capillary forces if the Ge pedestals became smaller.

In order to investigate the elastic strain distribution of the GeSn microdisks, μ-Raman was performed at RT on 5 μm diameter disks, using a 633 nm laser for excitation with a spot size of about 600 nm. The laser power is only 1% of the maximum power (15 mw) to minimize the thermal effects [34], and the penetration depth of the excitation laser in the GeSn films is approximately 50 nm [26]. The Raman shift was measured by fitting the spectra with Lorentzian functions.

Figure 5b shows the one-dimensional Raman line scans for Ge_0.902_Sn_0.098_ microstructures. It is clearly noted (1) for the 5 μm diameter mesa without undercut, the Raman peak associated with the Ge-Ge LO phonon mode shifts unobviously toward lower wavenumbers from the center to the edge of the mesa, and the large Raman shift indicates that there is a large compressive strain in the GeSn mesa; (2) for the 5 μm diameter microdisk with 2 μm diameter Ge pedestal, the Raman peak associated with the Ge-Ge LO phonon mode shifts obviously toward lower wavenumbers from the center to the edge of the disk, revealing a strain gradient due to anchoring to the comparatively large Ge pedestal. And the 5 μm GeSn microdisk with 2 μm diameter pedestal still maintains at its periphery a certain amount of compressive strain; (3) the Ge-Ge peak positions measured along the diameter of the 5 μm Ge_0.902_Sn_0.098_ microdisk with 300 nm diameter Ge pedestal remain consistent and have an obvious reduced Raman shift. Therefore, the disks with narrow Ge pedestals (about 300 nm diameter) are expected to be fully relaxed.

**Fig. 6:**
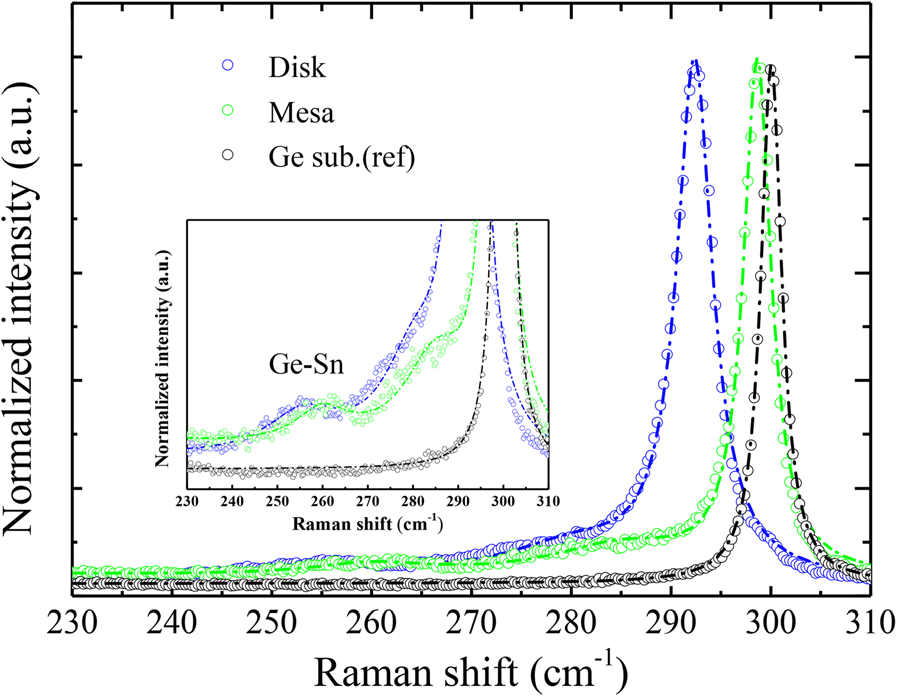
Comparison of the Raman spectra of the 5 μm Ge_0.902_Sn_0.098_ microdisk with 300 nm diameter Ge pedestal, 5 μm Ge_0.902_Sn_0.098_ diameter mesa and a bulk Ge substrate as a reference. Inset: enlarged view of the Raman spectra. The Ge-Sn peaks can also be observed around 260 cm^−1^, and down shift with decreasing the diameter of Ge pedestals

In Fig. 6, we present the typical Raman scattering spectrum of 5 μm diameter Ge_0.902_Sn_0.098_ microdisk with 300 nm diameter Ge pedestal. For the disks, the Raman spectra show an asymmetric peak at a frequency of 292.4 cm^−1^ corresponding to the Ge-Ge LO mode. The Raman spectra as recorded for the 5 μm diameter mesa and the reference bulk Ge (001) substrate are also plotted for comparison. Note that after undercutting, clear Raman shift (− 6.3 cm^−1^) of the Ge-Ge peak can be observed for 9.8% Sn microdisks, since the Raman frequency shift in semiconductor alloys is influenced mainly by strain and composition. Previous Raman studies [33, 35–37] have shown that the Raman shift of the Ge-Ge LO phonon mode in GeSn can be expressed as a function of the Sn concentration *x*_Sn_ and the in-plane biaxial strain *ε*_∥_ following Eq. (1):

1 $$ \Delta \omega ={\omega}_{\mathrm{Ge}\mathrm{Sn}}-{\omega}_{\mathrm{Ge}}=\mathrm{A}\cdotp {x}_{\mathrm{Sn}}+\mathrm{B}\cdotp {\varepsilon}_{\parallel } $$

where *ω*_GeSn_ and *ω*_Ge_ are the peak position of the Ge-Ge LO phonon mode in GeSn and in bulk Ge, respectively, while A and B are coefficients.

The Ge-Ge LO Raman peaks for the mesa and disks show the shift of − 1.4 cm^−1^ and − 7.7 cm^−1^ compared with the bulk Ge peak (300.1 cm^−1^). By taking the coefficients from previous experimental results [37], the value of the in-plane biaxial stain *ε*_∥_ is calculated to be − 1.18% for the as-grown GeSn mesa. For the fabricated microdisks, the in-plane biaxial stain *ε*_∥_ is approximately equal to 0, confirming the almost completely strain relaxation of the microdisks. According to the recent theoretical calculation of Ge_1-*x*_Sn_*x*_ electronic band structure [38], the as-grown Ge_0.902_Sn_0.098_ layer is an indirect bandgap material with bandgap energy about 0.56 eV, while the fully relaxed Ge_0.902_Sn_0.098_ microdisk is a direct bandgap material with bandgap energy of 0.50 eV.

## Conclusions

In conclusion, the GeSn microdisks were successfully fabricated by combining selective epitaxial growth with selective wet etching process. HRTEM and SEM were performed to confirm that the GeSn alloy is highly crystalline and that the GeSn microdisk structure is readily formed by a simple selective wet etching. The μ-Raman measurements reveal that the strain relaxation of GeSn microdisk will be larger with decreasing the diameter of Ge pedestal due to the simultaneously decreasing mechanical constraint from the smaller Ge pedestal. And finally, the high quality and completely strain relaxation GeSn microdisks were achieved by this more cost-effective method. The fabrication process is also a very promising method to achieve smaller GeSn mesa size until lateral quantum size effect is getting important, and to obtain other GeSn nanostructures such as GeSn quantum dots, GeSn nanomesh, and GeSn nanowires for future Si-compatible photonic and electronic device applications.

## Data Availability

The data that support the findings of this work are available from the corresponding author upon reasonable request.

## Abbreviations

APM: Ammonia peroxide mixture (wet etchant)
FFT: Fast Fourier transformation
HRTEM: High-resolution transmission electron microscopy
MBE: Molecular beam epitaxy
PECVD: Plasma-enhanced chemical vapor deposition
RT: Room temperature
SEM: Scanning electron microscopy
SIMS: Secondary ion mass spectrometry
μ-Raman: Micro-Raman spectroscopy
